# Accuracy of Psychiatrists’ Assessment of Medication Adherence in an Outpatient Setting

**DOI:** 10.7759/cureus.11847

**Published:** 2020-12-02

**Authors:** Katherine B Martin

**Affiliations:** 1 Psychiatry, Lehigh Valley Health Network, Allentown, USA

**Keywords:** clinical psychiatry, bipolar disorder, psychopharmacology, nonadherence, schizophrenia, schizophrenia and other psychotic disorders

## Abstract

Background: Many patients have uncontrolled psychiatric symptoms because they are not taking their medication as prescribed. Psychiatrists may have difficulty accurately assessing medication adherence, which is important because it helps guide them in how they prescribe. If nonadherence is the cause of uncontrolled symptoms, then strategies to improve adherence are advised. However, if nonadherence is not the cause, then the usual course of action would be to intensify or modify the medication regimen. Knowing whether nonadherence is a factor at the time of an appointment could help guide clinical decision making in real-time.

Methods: A cohort of established patients in an outpatient mental health treatment clinic at a large health network was studied from November 2018 to August 2019. Blood drug levels of several oral antipsychotic medications were obtained and placed in the following three categories: below, within, or above the therapeutic range of published cutoff points. Treating physicians answered Likert-scale questions regarding their assessment of patient adherence. Subsequently, blood drug levels were compared to the psychiatrists’ assessment of adherence using a Kappa coefficient.

Results: Sixty-four patients being prescribed antipsychotic medications were analyzed. A total of 87.5% of treating psychiatrists thought their patients were always adherent or adherent greater than 50% of the time. However, based on blood levels, 14% of the 42 patients at target FDA dosing for their medication and diagnosis were below the therapeutic range. The Kappa coefficient was used to find the level of agreement between the treating psychiatrist’s perception of patient adherence and the blood drug level. It was determined to be 0.14 which is consistent with no agreement between the two measurements.

Conclusions: Treating psychiatrists inaccurately estimated oral antipsychotic medication adherence based on clinical impression alone. Making an objective measure of adherence available at the time of an appointment could help psychiatrists recognize nonadherence in real-time and inform prescribing decisions.

## Introduction

Nonadherence with oral antipsychotic medications is commonly cited as a cause of partially to fully uncontrolled psychiatric symptoms [1,2]. Treating psychiatrists should make decisions in real-time about how to best handle such symptoms. They should consider if the cause of the symptoms is related to nonadherence versus lack of efficacy so that they can best decide whether to increase the dosage of a medication or change the medication. It remains questionable and unclear how accurate psychiatrists are about predicting whether their patients are adherent based on clinical assessment alone. However, a prior study demonstrated that psychiatrists in an emergency room setting were not accurate in predicting whether patients were compliant with their antipsychotic medications [3]. Additionally, in studies with other disciplines, primary care physicians were not accurate at predicting adherence to blood pressure medications [4]. This study sought to assess how accurate treating psychiatrists were at identifying their patients’ medication adherence by comparing their clinical impression with blood medication level in an outpatient setting.

## Materials and methods

The goal of the study was to retain blood samples from individuals receiving Risperidone, Aripiprazole, Clozapine, Olanzapine, and Quetiapine therapy for research purposes in the development of the company’s diagnostic tests. Patients at the institution’s outpatient psychiatric practices who were being prescribed the psychotropic drugs of interest-based on a review of their electronic medical record were approached to see if they would be willing to have their blood drawn. Patients were compensated with a $25 gift card for their participation. Saladax Biomedical, Inc. performed the analysis of medication drug levels using their validated immunoassays, and results were obtained within several hours. Subsequently, treating psychiatrists of the enrolled patients were asked to fill out a two-question survey based on a prior study by Meddings et al. regarding their perception of their patient’s adherence to the medication as well as how difficult the lack of adherence made it to control their patient’s symptoms [4].

Descriptive statistics were used to summarize the study sample. The two survey questions were summed to create a continuous adherence score, after reverse-scoring the first survey question, “How often does this subject adhere to the psychiatric medication regimen?” Psychiatrist perception of patient adherence was defined by using responses to the second survey question, “How much does a lack of adherence make it difficult to control this subject’s symptoms?” If the physician responded to this survey question as “A lot” or “A great deal” the patient was considered non-adherent. The Chi-square test was used to determine if there was an association between physician perception of adherence and therapeutic range. The Kappa coefficient was used to find the level of agreement between the psychiatrist’s perception of patient adherence and drug blood level. Statistical significance was set at p=0.05. Statistical analysis software (SAS, Cary, NC, USA) was used to conduct statistical analysis.

## Results

Sixty-four patients were included in the study. General demographics of the study participants are included in Table 1.

**Table 1:** Demographic and clinical characteristics for the entire study and stratified by drug IQR: interquartile range

	Total (n=64)	Risperidone (n=13)	Aripiprazole (n=10)	Clozapine (n=13)	Quetiapine (n=17)	Olanzapine (n=11)
Age, years median (IQR)	55.0 (46.0-60.0)	59.0 (57.0-67.0)	49.5 (45.0-56.0)	54.0 (48.0-60.0)	56.0 (47.0-59.0)	50.0 (44.0-59.0)
Gender						
Male	35 (54.7)	11 (84.6)	2 (20.0)	5 (38.5)	9 (52.9)	8 (72.7)
Female	29 (45.3)	2 (15.4)	8 (80.0)	8 (61.5)	8 (47.1)	3 (27.3)
Patient’s diagnosis						
Bipolar	11 (17.2)	1 (7.7)	3 (30.0)	0	5 (29.4)	2 (18.2)
Bipolar 2	3 (4.7)	0	2 (20.0)	0	0	1 (9.1)
Bipolar, depressed	10 (15.6)	1 (7.7)	0	0	7 (41.2)	2 (18.2)
Bipolar, mixed	7 (10.9)	2 (15.4)	1 (10.0)	0	3 (17.7)	1 (9.1)
MDD	1 (1.6)	1 (1.7)	0	0	0	0
Schizoaffective	19 (29.7)	6 (46.2)	3 (30.0)	6 (46.2)	2 (11.8)	2 (18.2)
Schizophrenia	13 (20.3)	2 (15.4)	1 (10.0)	7 (53.9)	0	3 (27.3)

Medication dosages for each patient were reviewed to assess whether the patient was at or below the target doing based on FDA prescribing guidelines for their diagnosis [5]. In instances when a dosing range was given, the minimum dose of the range was used as the target dose. This step was performed to ensure that low dosing was not the reason for a subtherapeutic blood drug level rather than nonadherence. For Risperdal, a minimum dose of 4mg for schizophrenia/schizoaffective and 1mg for bipolar disorder or major depressive disorder was required to be a target. For Aripiprazole, a minimum dose of 10mg for schizophrenia/schizoaffective and 15mg for bipolar disorder was required. For Clozapine, a minimum dose of 300mg for schizophrenia/schizoaffective was required. For Quetiapine, a minimum dose of 150mg for schizophrenia/schizoaffective, 300mg for bipolar depression, and 400mg for bipolar mania was required. For Olanzapine, a minimum dose of 10mg for either schizophrenia/schizoaffective or bipolar disorder was required. Using these FDA guidelines, 42 (66%) patients were at target dosing of their medications. There was no maximum limit imposed on dosing as the purpose was to ensure that low dosing was not the reason for subtherapeutic blood drug levels. See Table 2 for details.

**Table 2:** Dose range for each medication

	Minimum Dose (mg)	Maximum Dose (mg)	Average Dose (mg)	Below FDA Target Dose	At FDA Target Dose
Risperidone (n=13)	2.0	6.0	4.0	3 (23.1%)	10 (76.9%)
Aripiprazole (n=10)	5.0	30.0	16.5	2 (20.0%)	8 (80.0%)
Clozapine (n=13)	50.0	600.0	325.0	6 (46.2%)	7 (53.8%)
Quetiapine (n=17)	50.0	800.0	313.2	9 (52.9%)	8 (47.1%)
Olanzapine (n=11)	7.5	35.0	19.8	1 (9.1%)	10 (90.9%)

Sixty-four surveys were filled out by nine different treating psychiatrists at three different outpatient psychiatric practices within the health network. One participant had two surveys completed because he was on multiple psychiatric medications and provided two blood samples. One participant’s survey was not filled out because his psychiatrist practiced outside the network. Most treating psychiatrists thought that their patients were always or frequently adherent to their psychiatric medication regimen. Similarly, most treating psychiatrists did not find that a lack of adherence made it difficult to control their patient’s symptoms. See Table 3 for details.

**Table 3:** Physician survey results

	Total (n=64)	Risperidone (n=13)	Aripiprazole (n=10)	Clozapine (n=13)	Quetiapine (n=17)	Olanzapine (n=11)
How often does this subject adhere to the psychiatric medication regimen?						
Never	0	0	0	0	0	0
Rarely, less than 50% of the time	1 (1.6)	1 (7.7)	0	0	0	0
Occasionally, about 50% of the time	7 (10.9)	0	1 (10.0)	0	5 (29.4)	1 (9.1)
Frequently, more than 50% of the time	24 (37.5)	7 (53.9)	5 (50.0)	2 (15.4)	6 (35.3)	4 (36.4)
Always	32 (50.0)	5 (38.5)	4 (40.0)	11 (84.6)	6 (35.3)	6 (54.6)
How much does lack of adherence make it difficult to control this subject’s symptoms?						
Not at all	41 (64.1)	8 (61.5)	4 (40.0)	12 (92.3)	7 (41.2)	10 (90.9)
Some	15 (23.4)	4 (30.8)	3 (30.0)	0	8 (47.1)	0
Moderately	2 (3.1)	0	0	0	1 (5.9)	1 (9.1)
A lot	4 (6.3)	1 (7.7)	1 (10.0)	1 (7.7)	1 (5.9)	0
A great deal	2 (3.1)	0	2 (20.0)	0	0	0

For the 42 patients at target medication dosing based on FDA guidelines for their diagnosis, six (14%) were found to have blood drug levels below established cutoffs [6,7]. Based on these cutoffs the minimum blood level to be considered “therapeutic” was 20ng/mL for Risperdal, 100ng/mL for Aripiprazole, 350ng/mL for Clozapine, 100ng/mL for Quetiapine, and 20ng/mL for Olanzapine. Only the blood levels of the 42 patients at target medication dosing were used for comparison with the psychiatrists’ perception of adherence because patients on subtherapeutic dosing of the medication may have had a subtherapeutic level due to low dosing rather than nonadherence. 

Of the six patients with target medication dosing but subtherapeutic blood medication levels, five were thought to have been adherent by their treating psychiatrist. Of the 36 patients with target medication dosing and therapeutic blood medication levels, two were thought to have been nonadherent. Of note, adherence was defined as those patients who were thought to take their medication frequently or always. Non-adherence was defined as those patients who were thought to take their medication occasionally, rarely, or never. Using these definitions, there was no statistical difference between the two groups. See Table 4 for details.

**Table 4:** Physician perception of adherence and classification of the therapeutic range for patients at or above target dose

	Below Therapeutic Range (n=6)	At or Above Therapeutic Range (n=36)	p-Value
Non-adherent	1 (33.3)	2 (66.7)	0.3780
Adherent	5 (12.8)	34 (87.2)	

 

The Kappa coefficient was also determined for all drugs combined and for each individual drug. A Kappa coefficient measures the extent of agreement between two measures, which in this case was the physician’s perception of patient adherence (defined as being either adherent or non-adherent), and medication blood level (defined as below therapeutic range or at/above therapeutic range). When looking collectively at all 42 blood drug levels, the Kappa coefficient was 0.14, which is consistent with no agreement between the two measures. When broken down by individual medication, only Risperdal showed almost perfect agreement. Olanzapine showed no agreement and Quetiapine showed minimal agreement. Aripiprazole and Clozapine could not be calculated because no patients fell into the non-adherent definition. See Table 5 for details.

**Table 5:** Kappa for patients at or above target dose ^a^The Kappa coefficient for Aripiprazole could not be calculated because no patients fell into the non-adherent definition. ^b^The Kappa coefficient for Clozapine could not be calculated because no patients fell into the non-adherent definition.

Drug	Kappa (95% CI)
All drugs combined (n=42)	0.14 (-0.24 to 0.52)
Risperidone (n=10)	1.0 (1.0–1.0)
Aripiprazole (n=8)	N/A^a^
Clozapine (n=6)	N/A^b^
Quetiapine (n=8)	-0.20 (-0.49 to 0.09)
Olanzapine (n=10)	-0.11 (-0.26 to 0.04)

## Discussion

The overall findings suggest that treating psychiatrists were not accurate at determining adherence to oral antipsychotic medication based on clinical examination alone given the low Kappa coefficient (0.14). This finding could provide support for the need for point-of-care testing of blood medication levels to help with timely prescribing decisions.

It is notable that most treating psychiatrists in this study thought that their patients were adherent. Several factors could explain this finding. The study participants were chronic, established, outpatients. Most of the psychiatrists had been treating their patients for at least 5 to 10 years. To remain enrolled in the outpatient practices, patients must be compliant with appointments or they are at risk of having their case closed. This expectation alone would suggest some basic level of adherence with treatment. Also, the study was voluntary, and patients had to agree to participate, which would also suggest a more compliant population.

With all the features of this study population, it might have been expected that treating psychiatrists would have had a good ability to assess adherence. However, this was not the case. Therefore, these findings would suggest that other settings, such as the emergency room or inpatient psychiatric units where patients are not known to the treating psychiatrists, would be even more challenging environments to assess adherence and make accurate prescribing decisions. These environments would likely benefit even more strongly to access to point-of-care testing of antipsychotic drug levels.

There are several limitations to this study. The most notable is its small sample size. Only the 42 samples from patients who were at target dosing for their medication and diagnosis could be utilized. If all samples had been included, then the results may have been confounded by low dosing rather than nonadherence as blood medication levels are dose-dependent. An additional limitation is the assumption that knowing whether a patient is an adherent will help with symptom control. For example, some patients will still exhibit symptoms even while on medication, and some symptoms, such as negative symptoms, are not as responsive to medications. Further studies would be needed to see if timely access to blood medication levels impacts treatment outcomes.

## Conclusions

Treating psychiatrists in an outpatient setting were not accurate in determining oral antipsychotic medication adherence based on clinical impression alone. Making an objective measure of adherence available in real-time during a patient encounter may help providers decipher whether symptoms are a result of nonadherence versus ineffectiveness and help to inform prescribing decisions.
